# Fusing strategic risk and futures methods to inform long-term strategic planning: case of water utilities

**DOI:** 10.1007/s10669-021-09815-1

**Published:** 2021-05-25

**Authors:** Ana Luís, Kenisha Garnett, Simon J. T. Pollard, Fiona Lickorish, Simon Jude, Paul Leinster

**Affiliations:** 1 School of Water, Energy and Environment, Cranfield University, College Road, Cranfield, MK42 0AL Bedfordshire UK; 2 Empresa Portuguesa das Águas Livres S.A., Grupo Águas da Portugal, Avenida de Berlim 15, 1800-031 Lisbon, Portugal

**Keywords:** Water, Utility, Risk, Futures, Strategic decisions

## Abstract

Risks and futures methods have complementary strengths as tools for managing strategic decisions under uncertainty. When combined, these tools increase organisational competency to evaluate and manage long-term risks, improving the flexibility and agility of the organisation to deal with gross uncertainties. Here, we set out a framework to guide the assessment of strategic risks for long-term business planning, based on its application at Portugal’s largest water utility, Empresa Portuguesa das Águas Livres. Our approach extends strategic risk assessment by incorporating scenario planning—a futures approach used to help the utility move beyond single point forecast of risks to focus on critical dimensions of uncertainty that are fundamental to the resilience of corporate objectives and their vulnerability to external pressures. We demonstrate how we combine two complementary approaches—risk and futures—and use them to assess (i) how a set of baseline strategic risks for a water utility evolves under alternative futures, (ii) the aggregate corporate-level risk exposure, and (iii) the process and responses needed to manage multiple, interdependent strategic risks. The framework offers a corporate approach to evolving strategic risks and improves a utility’s (i) knowledge of uncertainties, (ii) ability to assess the impacts of external developments over long time horizons and the consequences of actions and (iii) degree of flexibility to adapt to possible future challenges. The framework supports risk managers in their long-term strategic planning, through the appraisal and management of multiple, interdependent long-term strategic risks and can be replicated in other organisational contexts to bridge operational and corporate perspectives of enterprise risk.

## Introduction

Water utilities are capital-intensive organisations that operate extensive and geographically dispersed assets in order to provide safe, wholesome and affordable drinking water that has the trust of customers. In doing so, they manage a vast portfolio of risks preventatively and plan asset maintenance and replacements for the long-term, usually over three or four decades, because of their huge investment requirements.

‘Master planning’, the periodic, high-level infrastructure plans that utilities produce for their operations, has an established history within the water sector. These long-term appraisals seek to justify, assemble and prioritise cases for investment in the utility’s asset base and for broader environmental protection, usually by reference to customers’ willingness to pay for improvements. In updating their master plans, utilities have needed to accommodate ‘changing baseline risks’ when advancing their longer-term plans to politicians, regulators and investors. The dynamic nature of a utility’s baseline risk is characterised by a complex mix of uncertain factors (e.g. population demand, land use change, climate change, fluctuating investor confidence and regulatory change) whose impacts are not well understood even though currently identifiable. Such factors can be disruptive to the corporate risk portfolio and can be overwhelming for risk managers, especially as the pace of change is making disruptive events (e.g. floods, pandemics) more likely over time. ‘Risks’ and long-term ‘futures’ are, therefore, important components for the strategic decisions that water utilities make, especially if they are to maintain infrastructure, systems and processes that ensure resilience across the business.

The authors recognise that a utility’s strategic risks need to be understood and planned for by considering its baseline risks in a landscape of long-term challenges. Using critical reviews, in-company ethnographic studies and semi-structured interviews with risk ‘owners’ from a sustained network of international utilities, some of the authors have (i) illustrated the use of risk ‘heat maps’ with horizon-scanning methods, projecting changes to a set of strategic risks forward in time to inform discussions about water utility resilience (Prpich et al. 2011; Luís et al. 2015, 2016) and (ii) analysed trends in the nature of risks managed over a 10-year period, revealing how risks have become progressively extrinsic in nature (Chalker et al. 2018).

Despite these developments, a gap remains for integrated frameworks and processes to guide the assessment of strategic risks for long-term business planning. For the decision science community, the question of whether *utilities can inform their long-term master planning exercises with dynamic business risk knowledge* is of interest. This requires investigating (i) the relationship between business risk portfolios, (ii) what the triggers of risks are, the conditions and stimuli, (iii) the different external factors involved and their interdependencies, and (iv) how those triggers change, the extent of change and the consequences for the business (e.g. long-term investment plans). This research question is the subject of this paper and our interest is in advancing a methodological approach that allows risk managers to do so.

## Risks, scenarios and futures

Existing probabilistic-based risk management approaches are recognised as being unreliable with regards to unexpected low frequency hazard events (e.g. combined events) and complex systems with a number of sub-units that have a high degree of dynamic interaction and emergent behaviour (e.g. non-linearity, ‘scale-free’ behaviour). Thus, such approaches used in isolation are of questionable value in improving the resilience of utilities that face systemic, long-term risks—defined as trends or events that occur suddenly and are characterised by uncertainty in terms of the likelihood of the risk and its potential impact (Cinner and Barnes 2019). Fundamentally, the challenge for utility managers lies in connecting their operational risk management process to their strategic (long-term) planning process, where fragmented procedures for identifying and managing risks has had a negative impact on their level of preparedness and ability to be resilient to disruptive change.

The growing complexity of business decisions has tested our ability to model the interactions between drivers that *directly* affect business strategy (e.g. competitors, customers, suppliers) and those that *indirectly* affect the macro business landscape and that are often extrinsic to it (e.g. economy, politics, regulation; Vecchiato 2012, 2015; Rohrbeck and Schwarz 2013). For example, the multitude of influences on flood and drought management strategies has prompted research into the combination of risk assessment and scenario modelling (Lehner et al. 2006; Lane et al. 2011). Here, the consideration of uncertainty has been addressed by simulating the future; extrapolating past trends into the future, assuming some continuity of change. Numerical simulations of the future provide the basis for developing a mitigation strategy, but its effectiveness is reliant on how close the ‘real’ future is to the one simulated. Similar static assessments are made in risk analysis, where models have a baseline projection that is varied to derive scenarios of, say, high, medium and low risk.

The complexity of a utility’s decisions makes it difficult to produce a probabilistic estimate that offers a quantified confidence level that a decision is safe or optimal. We have suggested a more preventative and anticipatory approach to risk and opportunity ensures utilities are resilient to threats and disruptive change, while equally open to opportunities (Pollard et al. 2013). Here, we argue that improving a utility’s business resilience requires integrating multiple decision tools that draw on futures and risk management approaches to better understand and respond to strategic risks. We propose a ‘semi-quantitative’ approach to assess a utility’s strategic risks, and incorporate the use of alternative future scenarios to ‘evolve’ those risks over time, examining how sensitive risk management controls or ‘barriers’ are to a more ‘dynamic’ risk portfolio.

The *fusion of risk and futures methods* supports decision-making under uncertainty, emphasising how the treatment for emerging threats and the exploitation of future opportunities can help water utility managers explore the potential value of strategic flexibility. Generally, the risk analysis and futures practitioner communities have developed parallel, but not intersecting trajectories. They may be aware of one another’s ‘toolboxes’, but rarely are they combined and deployed together. Here, we focus on the potential for using alternative future (qualitative) scenarios to refine consideration of a set of baseline risks over longer-term planning horizons. The scenarios allow for a deeper exploration of combined uncertainties and consideration of disruptive change, thereby addressing deficiencies in static risk analysis that rely on probabilistic estimates of single future events.

**Fig. 1:**
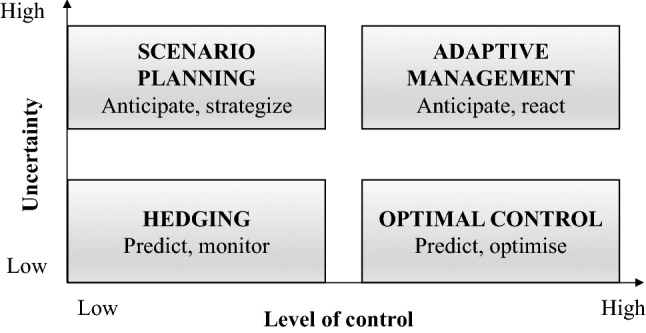
Role of scenario planning in corporate management (after Peterson et al. 2003)

Scenario planning offers a method for considering possible risks under a range of alternative futures, and to examine trade-offs associated with different strategic options whilst integrating a range of factors, such as physical, regulatory and financial issues, into decision-making. Scenarios are defined as a set of plausible, sequentially linked events that might potentially occur in the future (Jarke et al. 1998). These ‘world views’ are neither predictions nor forecasts of future events, but reflect the qualitative knowledge and assumptions about key relationships and driving forces, gathered through workshops or interviews with key experts and stakeholders (Amer et al. 2013; Tourki et al. 2013). Rather than establishing actions based on historical outcomes, scenarios encourage risk managers to consider unexpected events and examine the implications of trends from multiple perspectives to clarify current management actions in the light of plausible futures (Swart et al. 2004; Parson 2008; Durance and Godet 2010). The goal, therefore, is to understand the potential impacts of alternative scenarios on current management actions, rather than to select a desired outcome of an expected future (Fauré et al. 2017) or prioritise a range of scenarios on the basis of their degree of likelihood and influence (Karvetski et al. 2011). Several academic reviews exist in the foresight literature on scenario development (Bradfield et al. 2005; Varum and Melo 2010; Kuosa 2011; Saritas and Nugroho 2012; Amer et al. 2013; Tourki et al. 2013). One can see the value of scenario planning in the context of increasing extrinsic risk if one considers the level of control managers perceive over elements of uncertainty within their business environment (Peterson et al. 2003; Fig. 1).

Both scenario planning and adaptive management seek to address high uncertainty in the business environment (Fig. 1), but scenario planning aims to identify a course of action that is resilient to a range of alternative futures when management control is low, whilst adaptive management aims to implement predetermined actions depending on how the future unfolds. As such, adaptive planning is more suitable at the operational level where there are greater opportunities to make low risk trade-offs, whereas scenario planning is more suited to high risk decisions, such as long-term infrastructure investments where uncertainties are high (Peterson et al. 2003; Scott et al. 2012). This rationale underlines its value for long-term decision-making where external factors influence the decision outcomes. Indeed, this is often the case where extrinsic risks beyond the control of utility operators is of importance.

Given the above, scenario planning has been widely used in capital-intensive industries, such as the aerospace and petroleum industry, to carry out systemic analyses of change as uncertainty increases (Vecchiato 2012, 2015; Amer et al. 2013; Rohrbeck and Schwarz 2013). In the case of water utilities, scenarios have been used on occasion to inform strategic planning, focussing on isolated factors such as climate change, but rarely on multiple issues that, in combination, reflect higher levels of uncertainty (e.g. simultaneous change in environmental, economic, legislative and societal factors) (Means et al. 2010; Cosgrove 2013). The effective use of scenarios for strategic planning hinges, therefore, on the ability to understand the critical dimensions of uncertainty affecting the business (Fig. 1). This is essential to producing credible scenarios and better strategies to position the organisation for future success (Means et al. 2005). Indeed, the real value of scenario planning is when actions or measures to maintain strategic flexibility increase the organisation’s performance and thereby create new value—e.g. competitiveness, resilience (Miller and Waller 2003).

## Fusing strategic risk analysis and scenario planning tools in practice

Strategic risk analyses enable managers to assess the impacts of, and tolerance for, identified risks. Scenario planning on the other hand enables risk managers to consider the risk landscape, examine how risks evolve in the future and to identify the potential impacts of critical risks and thereby suitable responses. Both tools are essential for increasing organisational competency to manage strategic risks, improving the strategic flexibility and agility of the organisation, allowing risk managers to better assess the consequences related to strategic decisions.

### Limitations of traditional risk assessment approaches

A challenge for water utilities is identifying those uncertain events that might occur which cannot be fully identified through conventional risk practices alone, making disruption inevitable (Brown et al. 2017). Traditional risk models (e.g. risk matrix, fault tree analysis) are often developed based on the assumption of ‘closed or simplified systems’ (Ferdous et al. 2010), which tend to omit non-linear relationships in the form of interdependencies and feedbacks, non-linear dynamics and thresholds that give rise to trade-offs and unintended consequences that are more common in open systems (Liu et al. 2007).

Risk analysis provides a single point forecast of individual risks (e.g. the likelihood of pesticides contaminating a water body and impacting consumer health), which is effective in capturing the interactions between events, and allowing the implementation of detailed probabilistic (quantitative) risk assessments (Lindhe et al. 2012, 2009). These, however, have limited capability to assess systemic risks—i.e. the interaction between physical risks and ‘broader risk areas’, such as financial and reputational risks—and their potential escalation from operational to corporate levels in the utility.

One way to address this limitation is to build systemic diagrams where the interdependencies between events, exposures and harms, associated with different risks, are taken into consideration. These systemic models are, however, developed with reference to a single point in time and often do not do consider the dynamic and interrelated nature of external drivers that shape risk events. In striving for resilience, water utilities need to, *complement risk analysis with scenario planning* as it provides cross-case comparisons from disruptive events that could impact multiple sectors, and provide an investigation of the ‘unknown’ at system scale instead of individual parts (LRF 2015; Linkov and Palma-Oliveira 2017).

### Complementarity between risk and scenario tools

Significant literature exists on scenario typologies and the processes and techniques for building scenarios (Bradfield et al. 2005; Saritas and Nugroho 2012; Amer et al. 2013), but little is formally recorded about the practical ‘hands-on’ experience of using scenarios to ‘stress-test’ a company’s risks and the long-term benefits related to greater preparedness and increased competitiveness (O’Brien 2004; Varum and Melo 2010). In practice, it seems few companies systematically integrate qualitative scenarios and simulation into their planning processes due to a fear of the unknown, lack of time, or adequate training in scenario planning techniques (Lemmens and Munsters 2007) combined with doubts about securing a return on investment (Rohrbeck et al. 2013).

Commentators have highlighted that scenarios have the value of initiating conversations about the business environment and enhancing the strategic thinking of managers (e.g. Brummell and MacGillivray 2008; Amer et al. 2013). Rohrbeck (2012) suggested that questions about value creation have been particularly relevant in the corporate context, where futures research has remained on the side line and not integrated well with operational and strategic management. Rohrbeck and Schwarz (2013) evaluated the value created from forward-planning (futures) activities for 77 multinational companies that: (1) gained insights about potential changes to their operating environment; (2) responded positively to change by coordinating business objectives and strategic actions; (3) shaped the future by influencing other actors; and (4) facilitated organisational learning.

Rohrbeck and Schwarz’s (2013) observations have parallels in corporate risk management, in that the ‘process’ itself is often as important as the strategies produced (Wack 1985; Koivisto et al. 2009; Amer et al. 2013). Corporate risk analyses allow managers to: (a) open their mindsets to better understand the aggregate risk to the corporate objectives, (b) compare the aggregate risk against to the utility’s risk appetite, tolerance and capacity through knowledge exchange, and c) manage high-level risk metrics that alert the Executive to emerging risks and so enhance preparedness for change (Allan et al. 2013; Schiller and Prpich 2014). Koivisto et al. (2009) highlighted the commonalities between risk and scenario approaches, further developed by Luís et al. (2016) in the context of water utilities (Table 1).

**Table 1:** Complementary steps in risk and scenario analysis

Approaches	Risk analysis	Scenario analysis
Purpose	Strategic planning	
Development phases	(1) problem formulation (2) risk evaluation (3) risk acceptation (4) options appraisal (5) risk management	(1) focal question (2) scenario building (3) implications of the scenarios (4) strategic actions appraisal (5) uncertainty management
Methods used	Quantitative, semi-quantitative or qualitative methods	
Knowledge making process	Factual evidence, explicit knowledge of experts and public perception incorporated to broaden the basis of knowledge and values that underpin decision-making	
Benefits	Opening “mental maps” and helping to initiate new conversations among the different actors at the utility, city and basin levels	

### A framework for integrating strategic risk and scenario analyses

To visualise risks over the long-term, there is a need to consider the interdependent and systemic nature of strategic risks (Luís et al. 2015), and an assessment of how these risks evolve under a range of alternative futures, shaped by a set of drivers of change (Luís et al. 2016). Herein lies the potential for combining scenario planning to help utility managers move beyond single point forecasts of risks to focus on the most critical dimensions of uncertainty that are fundamental to the resilience of corporate objectives and their vulnerability to external pressures (Swart et al. 2004; Means et al. 2010). Incorporating the use of alternative future scenarios supports the development of flexible strategies that can cope with changing baselines and alternative outcomes. Therefore, in combining strategic risk and scenario analyses, we seek to inform long-term planning exercises with dynamic business risk knowledge (Luís et al. 2015, 2016).

**Fig. 2:**
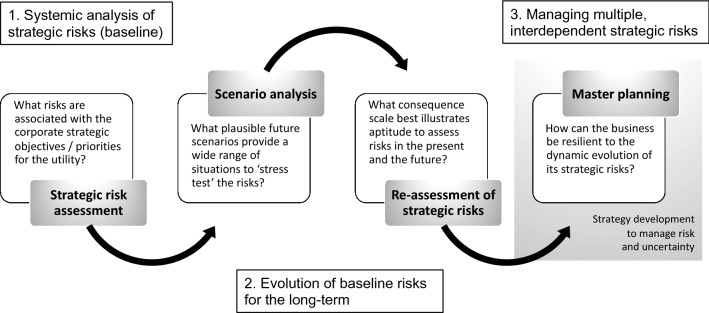
An integrated risk and futures framework

Our approach starts with an identification of the utility’s corporate objectives, at Board level, which is cascaded down to tactical and operational levels, where risk managers and risk experts carry out an analysis of the utility’s strategic risks, using risk assessment tools to consider the interdependent and systemic nature of the strategic risks. These baseline risks are then ‘evolved’ under multiple envisaged alternative futures, allowing managers to assess the likelihood and consequences of the risks occurring in each scenario that reflects different assumptions about future developments (e.g. climate, demographic, economic and technological change). This assessment forms the basis for determining the overall portfolio risk exposure, allowing risk managers to identify future threats and opportunities and devise strategies for master plans (Fig. 2).

Below, we outline our framework for combining strategic risk and futures analysis (Fig. 2), reflecting on its first application at the largest water utility in Portugal: Empresa Portuguesa das Águas Livres (EPAL).

#### Overview of EPAL

EPAL is the oldest and largest water utility in Portugal. Founded in 1868 as CAL—Companhia das Águas de Lisboa, a privately owned water company supplying Lisbon, it became a public limited company in 1974, and is now owned by Grupo Águas de Portugal, which is fully state-owned. The utility supplies water to approximately 3 million people (about a third of the Portuguese population). EPAL has approximately 700 staff and assets with a net value of about 900 million Euros.

EPAL operates a regional service system that assures bulk supply to 35 municipalities, north of the River Tagus. Its operations include the abstraction, treatment and transport of drinking water. EPAL also provides domestic water supply to Lisbon through the city’s distribution network. EPAL’s water supply system includes approximately 2100 km of water mains, 42 pumping stations, 40 water tanks, 25 chlorination points and around 100,000 service connections.

Over its 150 + years history, EPAL has faced differing challenges with each era posing new threats that the utility has had to address. The primary aim of creating EPAL was to supply drinking water to Lisbon through the 120 km extension of Alviela Aqueduct, thus solving Lisbon’s water supply challenges, moving forward. During its first century, key drivers of change facing EPAL included population growth and water quality improvements posing a challenge with droughts, floods and power generation failures. The need to respond to population change and water quality challenges necessitated enlarging system capacity with responses including the identification of new water resources, construction of new aqueducts and trunk mains during the 1930s, 1960s and 1980s, alongside new laboratory facilities. A major change in governance occurred during the 1970s with EPAL (CAL) shifting from a private concession to a state-owned company.

The early 2000’s saw EPAL facing a deficit of production capacity, especially in the summer, due to increased demand and water losses in its distribution network. This resulted in strategic decisions to enlarge the system’s capacity, in terms of drinking water production and transport, and to fight water losses. An extreme drought in 2005 had a major impact, resulting in a large-scale media campaign to change consumption habits, resulting in decreased consumption. With enlarged production capacity and decreased water losses, the period from 2007 has allowed EPAL to shift from prioritising investment in new assets to investing in addressing the complex intrinsic and extrinsic drivers of strategic change spanning political, social, economic, regulatory, issues, to asset management, risk management, information management and challenges including innovation and climate change. More recently, from 2010 onwards, EPAL has seen a shift in paradigm where the utility has transitioned from maintaining or increasing the efficacy of the service, in terms of water quantity, quality and reliability, to increasing the system’s efficiency, sustainability and resilience.

#### Systemic analysis of strategic risk: baseline

Decision analysts recognise that a ‘top-down’ and ‘bottom-up’ strategic risk assessment is required to capture interdependencies between different business units within organisations such as a water utility. We employed a ‘top-down/bottom-up’ approach to assess EPAL’s strategic risks. After working with the Board to define EPAL’s corporate objectives, these were then cascaded these down to tactical and operational levels for in-depth analysis of the events, exposure and harms to strategic risks, before escalating back up to the strategic level (i.e. the Board) for the results to be assessed. The process is summarised below (Table 2). A full account of the approach can be found in Luís et al. 2015.

**Table 2:** Comparison of strategic risks: baseline

Steps	Action	Methods and people involved	Output(s)
Corporate objectives identification	Define corporate values and priorities of different business units	Meeting with Board members and departmental executives	Corporate, strategic objectives
Events/exposure/harms systemic model	Assess and characterise events, exposures and harms to strategic risks; identify and characterise existing barriers that lower the likelihood and/or consequences of harms	Semi-structured interviews involving risk managers and experts from different departments related to each of the strategic risks	A systemic model, which captures the interdependencies between the risks and permits its visualisation to make it more accessible to Board members
Side by side risks comparison (baseline)	Evaluate and validate the likelihood of events, exposures and harms, as well as the aggregate consequences of harms, making comparisons of strategic risks	One-day workshop involving risk managers and experts from different departments related to each of the strategic risks	Narratives for each risk, validated systemic model and heat maps (for risk comparisons)

*Corporate objectives identification.* At EPAL, identifying the corporate baseline risks required first setting out the corporate values and priorities of different business units and transposing these into a set of ‘strategic objectives’, defined as the utility’s core objectives underpinning all departmental decisions (Keeney 1992). The process was carried out in a meeting involving cross-departmental discussion with decision-makers, at Board level, and with executives across departments to consider the full range of factors affecting its performance. A total of six strategic objectives were defined, aligned to common financial, regulatory and reputational risks at water utilities (Levinson et al. 2008; Morrison et al. 2010; Orr et al. 2011), including to guarantee: business sustainability, profitability, adequate water quantity and quality, reliability of supply and the business’ reputation and trust of customers and shareholders.

*Events/exposure/harm systemic model.* Next, we focussed on identifying the risks of not meeting the corporate objectives, defined as the ‘strategic risks’ (Frigo and Anderson 2011). Often in utilities strategic risks are compartmentalised within different business units, which makes it challenging for risk managers to monitor controls effectively, often missing multiple interconnected risks and their interdependencies in strategies developed. At EPAL, we combined strategic and operational risk assessments as a basis for determining the exposure of the overall risk portfolio. Following a ‘top-down’ assessment of the strategic objectives, we convened several brainstorming meetings with risk managers to carry out a ‘bottom-up’ assessment of ‘what they considered to be the strategic risks of EPAL’. This required a semi-quantitative assessment of the strategic risks, which was considered appropriate given the multidimensional nature of the risks. A full appraisal required mediating between operational, tactical and strategic risks, incorporating an analysis of the: (1) *events*, the root cause of activities defined temporally and spatially, (2) *exposures*, the pathways of impact from one or a number of events, and (3) *harms*, the direct impacts, effects or consequences resulting from the pathway(s) of exposure (Gormley et al. 2011). Risk managers were asked to appoint individual risk experts in their teams to assess the strategic risks via a number of semi-structured interviews (*n* = 12, ca. 2-h duration). Experts evaluated the systemic model to examine if any risks were missing and whether the interdependencies were well captured. They then moved to identify the likelihood of the events, exposures and harms, drawing on past studies at EPAL and on empirical knowledge (Waal and Ritchey 2007) to determine the likelihood and consequences of not meeting EPAL’s strategic objectives.

**Fig. 3:**
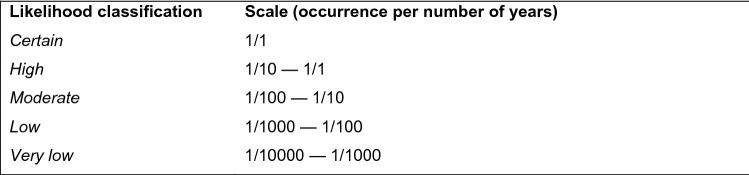
Likelihood classification (after Luís et al. 2015)

Luís et al. (2015) provides a comprehensive account of the approach. In summary, we applied a logarithmic scale to consider how likely the consequences of an EPAL-specific risk were to occur in the future (i.e. over 18 months from a base year of 2012). This is a common scale adopted for strategic risk appraisals (e.g. Andrews et al. 2003; FAO and WHO 2009). Assigning a numerical scale that showed the frequency of occurrence of a risk (Fig. 3) helped to reduce the level of ambiguity and lack of consistent interpretations of more qualitative probability phrases (e.g. likely, unlikely).

**Fig. 4:**
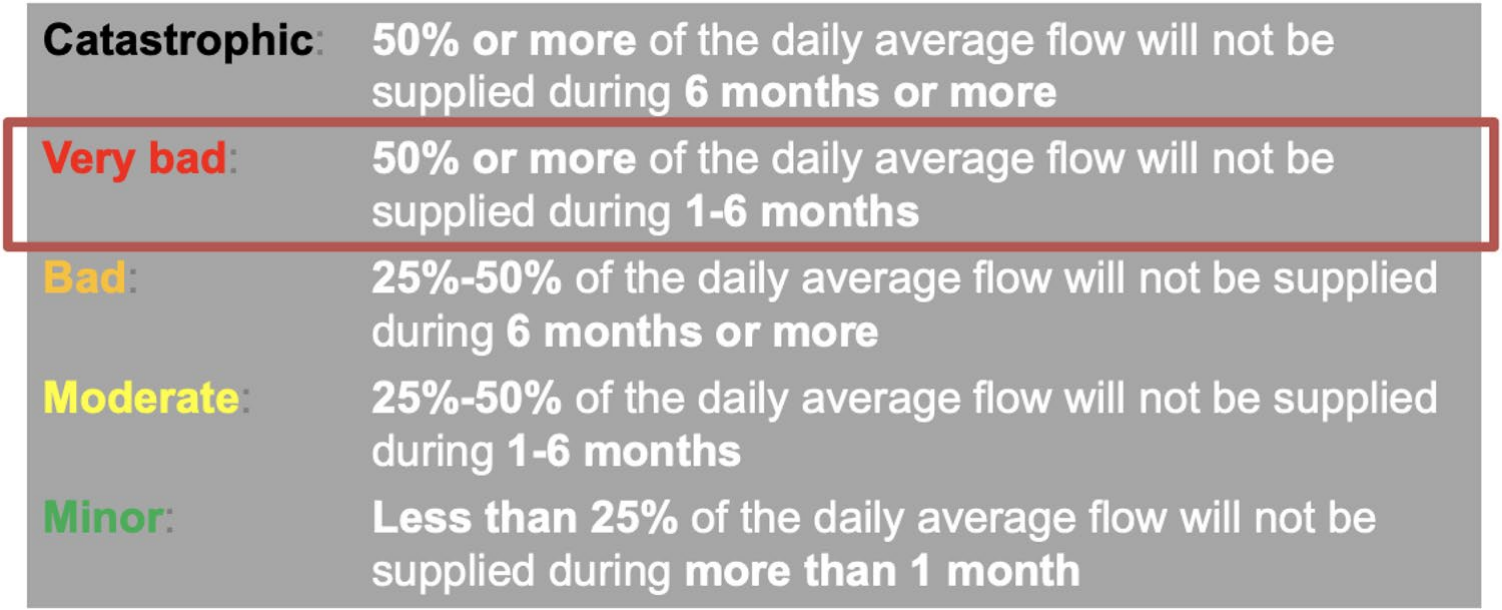
Consequence scale—water supply

A set of consequence attributes were selected to describe the impacts, including: (1) ‘type’, (2) extension (magnitude) and (3) duration (including irreversibility). We subsequently defined thresholds for these classes of consequences, ranging from catastrophic (the worst imaginable scenario) to minor impact. Taking water availability as an example, we asked “what is the plausible worst case scenario of a lack of water supply”? The speed at which EPAL was capable of responding to water supply challenges were considered; for example, a 6 months threshold took into consideration the estimated time to implement new abstractions or transfers from other water sources or transport systems (Fig. 4).

**Fig. 5:**
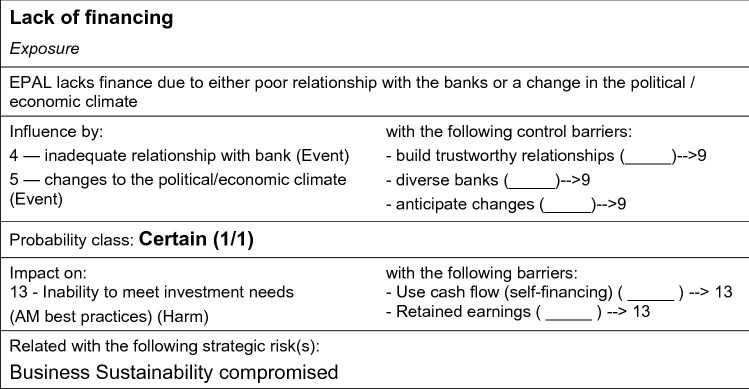
Triangulation of interview data—an example (after Luís et al. 2015)

The holistic model of the strategic risks was hence built through an iterative process, complemented by the identification and assessment of the performance of existing control barriers. During interviews, each risk expert was provided with the same systemic model and set of records characterising the events, exposures and harms and asked to comment on the (1) likelihood of events, exposures and harms, and (2) identification of existing barriers that mitigate exposures and harms and their respective consequences. Interview data were recorded in a similar format (Table 3) and triangulated (Fig. 5) and then compared to identify any inconsistencies and gaps in experts views, which was subsequently resolved through other rounds of expert interviews.

**Table 3:** Interview data records—an example (after Luís et al. 2015)

No	Box	Type	Evidences	Notes	Likelihood	Likelihood notes
2	Non-revenue from municipal clients	Event	Already happening (Torres Novas)	Municipal clients do not pay their water bills either because they cannot afford them or because they do not want to	Dpt. B/Dpt. C: 1/100—1/1000 Dpt. D: 1/10—1/100 (present economic context) Dpt. A: 1/1—1/10 (already happening)	Considering current economic climate (and associated financial impacts) in Portugal

Once the strategic risks were identified, attention was turned to assessing ‘how interrelated they were’ and the nature and extent of the impact on business performance. Interdependencies were characterised by a hierarchy of relationships, where we focussed on how risks from a specific business function or portfolio (e.g. micro or meso level) could affect the achievement of the strategic objectives at the corporate (macro) level. The assumption is that these interactions are “bi-directional” (Haimes et al. 2008) in that the activities and existing controls in a specific business unit of the utility will influence those in other units by way of interdependencies, which vary in strength, directedness and time scale (Wyrwoll et al. 2018).

**Fig. 6:**
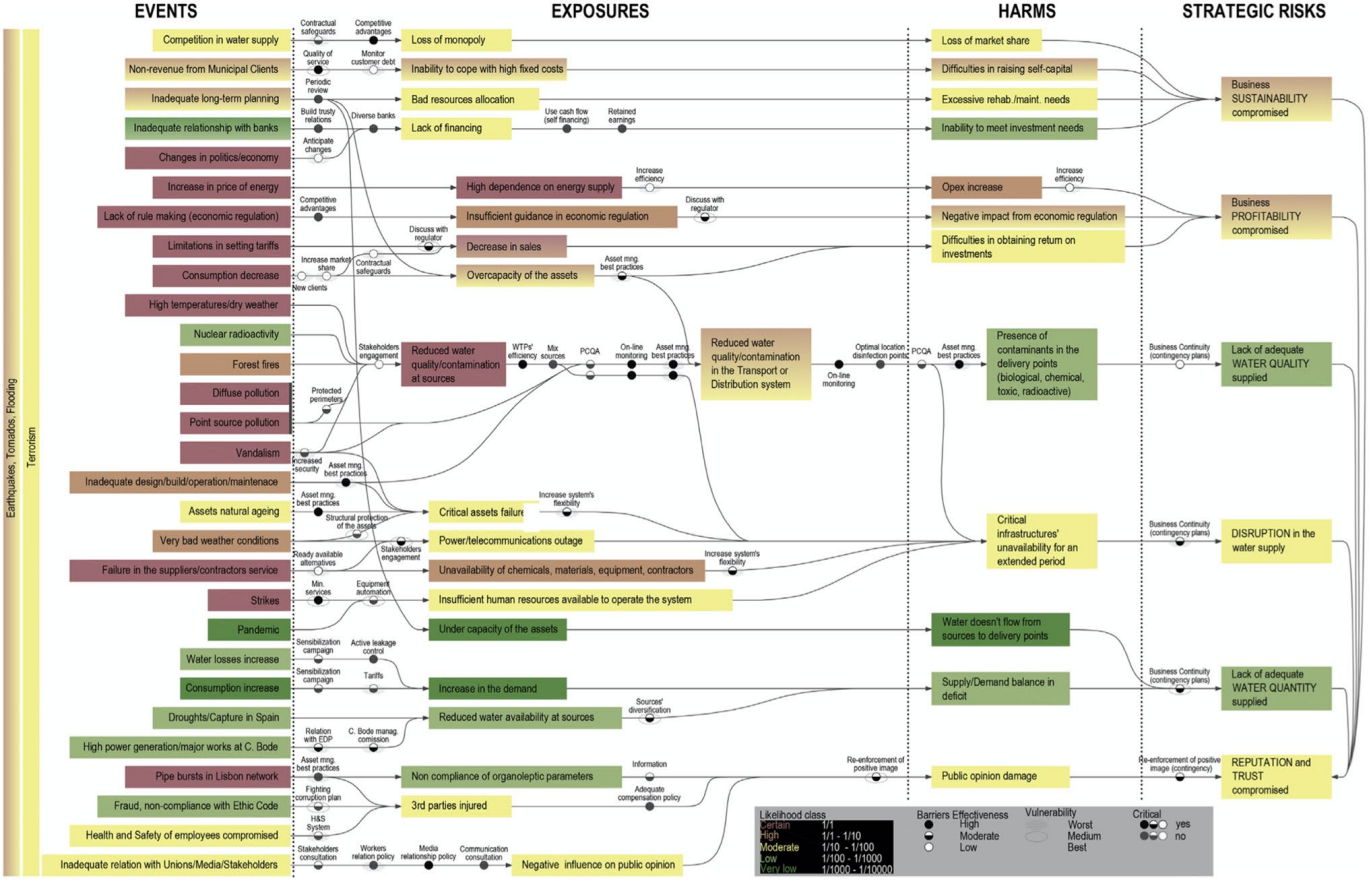
Event-exposure-harm systemic model—EPAL's baseline strategic risks (after Luís et al. 2015)

The output of this analysis was a systemic model that visualised the aggregate impact of multiple interdependent strategic risks (Gormley et al. 2011)—a useful visual that helped to gather insights about what drives the utility’s strategic risks. Figure 6 illustrates the interactions between the risks—with and without control barriers—and helped EPAL’s decision-makers to consider their performance (i.e. what controls are critical, vulnerable?). The model is colour-coded to illustrate the likelihood of events, exposures and harms, and enabled decision-makers to: (1) visualise the interactions between risks, (2) build an understanding of the risk probability—e.g. whether a risk had a naturally low probability of occurrence or if this was reduced due to existing barriers, and (3) review the efficacy of existing barriers and controls.

*Side by side risk comparison (baseline).* Next, we assessed the aggregate consequences of harms that allowed for comparing the strategic risks in a “heat map” (Prpich et al. 2013). This required first validating the risk evaluation in a one-day workshop with risk managers and experts from different departments at EPAL related to each of the strategic risks (*n* = 42). Validation focussed on evaluating the strength of existing control barriers, relying on expert knowledge to address questions about whether: (a) the analysis missed any existing barriers (if so, where), (b) how effective existing barriers are at mitigating risk to strategic objectives, (c) which barrier(s) are most critical, (d) which barrier(s) are most vulnerable, irrespective of their effectiveness, and (e) should there be additional barriers in the system?

Building on the systemic model, we compared the aggregate likelihood and consequences of the strategic risks in a “heat map” (Prpich et al. 2013) that allowed for visualising each strategic risk side by side, represented by an elliptical shape. This presented an alternative to the use of risk matrices that restrict risk classifications to ‘high, medium and low’, based solely on the likelihood and consequence assessed in isolation. In fact, underlying each ellipse there is the whole top/down, bottom/up assessment that led to the systemic model described above. This “heat map” increases our ability to reflect, through the length of the ellipses’ axes, the range of uncertainty the analysis embodies. This includes: (a) *aleatory* uncertainty that reflect the natural variability of the events (e.g. regulatory changes) and (b) *epistemic* uncertainty related to the lack of knowledge (e.g. demand changes) (Cox 2008). Bringing experts together allowed us to challenge individual biases, but we recognised the need to offset overall group bias. This was approached by reflecting on ‘uncertainty’ associated with the state of ‘evidence’ or the level of agreement/disagreement between risk experts (Fig. 4). We assigned the following criteria to reflect the level of uncertainty (adapted from Gormley et al. 2011):

- “Low”—there is empirical or scientific evidence,
- “Medium”—there is no empirical or scientific evidence, but there is a high level of agreement among experts,
- “High”—there is no empirical or scientific evidence and there is a low level of agreement among experts.

**Fig. 7:**
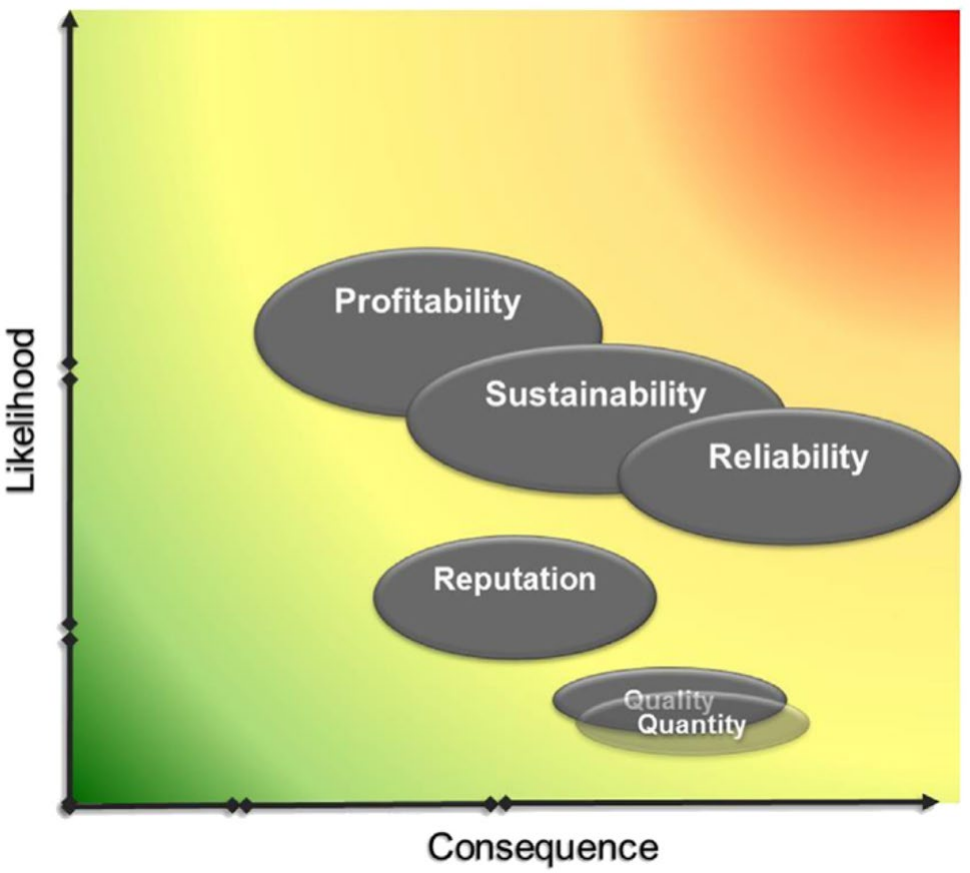
Heat map of EPAL’s baseline strategic risks (after Luís et al. 2015)

The elliptic shape of the risks in the heat map reflect a mix of aleatory and epistemic uncertainty through the size of the horizon and vertical axes where, for example, Fig. 7 shows this is far higher for the consequence than the corresponding likelihood. Figure 7 also shows that business sustainability, reliability and profitability are the risks with higher aleatory uncertainty in terms of their likelihood of occurrence. This may be due to the number of events that the company has no control of and as a result is difficult to predict. For example, change in regulation and economic stability often does not provide the stability needed for investment (Hecht et al. 2012) and may exacerbate business risk (Morrison et al. 2010).

The heat map allowed ‘side by side’ comparison of each strategic risk and supported communication with the Board as they included narratives on the character of the risk and the effectiveness of the current controls and barriers to manage the risks, drawing on information provided in the systemic model.

#### Evolution of baseline risks for the long-term

We employed alternative future scenarios to take account of multiple trends that may lead to different futures, rather than variations of a single future (Foster 1993), and used these to challenge the utility’s baseline risks over the next 30 years—the period of time for which the EPAL’s master plan is developed. Responding to external pressures requires building inter-organisational intelligence about (1) how a set of baseline risks could change given developments in the external business environment, and (2) what opportunities and threats need prioritising in long-term business plans. The process is summarised below (Table 4). A full account of the scenario approach can be found in Luís et al. 2016.

**Table 4:** Comparison of strategic risks over the mid to long term

Steps	Action	Methods and people involved	Output(s)
Key drivers and megatrends characterisation	Define broad drivers of change guiding strategic thinking about EPAL’s business performance over a 30-year period	PESTLE analysis to identify key drivers of change (including megatrends); informed by literature review and validated through expert elicitation (via workshop with EPAL’s technical experts/managers)	List of relevant key drivers and megatrends
Construction of future scenarios	Develop a consistent mix of drivers that define an alternative but plausible set of scenarios	Computer-aided cross-consistency analysis; synthesis of results to select scenarios	Four alternative scenarios, including narratives
Side by side risks (evolution) comparison	Stress-test the utility’s baseline strategic risks and assess the implications for achieving good strategic outcomes	Workshop involving risk managers and experts from different departments related to each of the strategic risks Re-evaluation of the likelihood and consequences associated with each strategic risk, against the different scenarios	Heat maps, showing comparative change in (evolution of) each strategic risk against a scenario

*Key drivers and megatrends characterisation* Luís et al. (2016) provides a comprehensive account of the approach. In summary, we employed morphological analysis (MA) (Ritchey 2011) to build the scenarios. This allowed for carrying out a rigorous investigation and definition of the relationships between numerous external and internal drivers of change, as a basis for achieving a high degree of differentiation in scenario configurations (Haines-Young et al. 2011). Researchers defined an initial list of key drivers—broad range of sector developments that could affect EPAL’s business performance—in a comprehensive desk-study using a PESTLE (i.e. Political, Economic, Social, Technological, Legislative and Environmental) analysis (Brown 2017). Megatrends—i.e. global, sustained macro-level developments—were derived from a 3-year longitudinal study of EPAL’s vulnerability to climate change (Jacinto et al. 2013; Grosso et al. 2012). Some megatrends with a narrow range of possible future developments were considered as ‘givens or predetermined’ (see Table 4) and assumed to be consistently occurring in all the scenarios.

A total of 12 key drivers (Table 5) were validated in a workshop with experts from different departments and with various management responsibilities in EPAL (*n* = 23). Experts were split into three groups based on their knowledge of the PESTLE themes: (a) social/economic (e.g. customer relations, financial and administrative, projects and works), (b) political/technology (e.g. trunks mains maintenance, information systems, projects and works, asset planning) and legislative/environmental (e.g. marketing, systems operations, water quality control, climate change). After an introduction to the drivers, participants worked in moderated groups to: (1) refine them by considering the ‘validity of the risks’ each driver poses to international water utilities, and (2) examine plausible change in the sector over a 30-year period, including abrupt change or disruptions (i.e. low probability, high impact events), to guide the identification of driver projections—i.e. the full range of plausible “states” each key driver could assume.

**Table 5:** Characterisation of key drivers/megatrends (after Luís et al. 2016)

PESTLE theme	Key driver/megatrend^a^	Description
Political	Organisational change	Relationships that EPA has with other utilities or businesses in the sector
Economic	Economic development/state of the economy	Growth of Portugal's economic output, defined as real GDP and average growth per year
Economic	Energy prices	Energy costs associated with water abstraction/sourcing, treatment and supply
Social	Population size/demographics^a^	Population growth (change) in the supply region
Social	Consumption patterns and environmental behaviour	Consumer lifestyles, attitudes towards the environment and their water consumption decisions
Technological	Infrastructure development	Infrastructural innovations and new infrastructure developments to address the deterioration and ageing of assets
Technological	Technology development	Technological developments and its implications (risks and opportunities) for water management strategies
Legal	Regulation and legislation (EU and national)	Legislation (i.e. National and European laws, directives and agreements) which shape water utilities’ regulations, management strategies and decisions
Environmental	Water quality	Water composition and level of sediments related to pollution
Environmental	Water availability	Average quantities of water in catchments that can be utilised by water utilities in the region
Environmental	Climate change^a^	Average rainfall, temperature and frequency of extreme weather events in the region
Environmental	Land use change^a^	Land use changes in the region

^a^Megatrend assumed as a given in all scenarios

*Construction of future scenarios* The process involved generating ‘a consistent mix of drivers’ (Ritchy 2011) in order to provide a *challenging set of futures* upon which to stress-test the utility’s baseline strategic risks and assess the implications for achieving good strategic outcomes. Building on outputs from the workshop, we employed the morphological analysis to carry out a pairwise comparison between every driver projection, whereby a judgement was made on whether a pair of projections can co-exist in a scenario (Ritchey 2011). Given the high number of pairwise combinations to be analysed (*n* = 474), we used Carma™software (Swedish Morphological Society http://www.swemorph.com/) to reduce the total set of driver configurations to a smaller set of internally consistent ones (Voros 2009; Ritchey 2011). The analysis generated a ‘morphological box’ (Fig. 6), where each pair of projections is resolved as either: (1) consistently a good fit, or best fit, or optimal pair, (2) ‘possible, could work, but are not optimal’, and (3) ‘impossible or very bad idea’ (Ritchey et al. 2002). The software deduced consistent relationships by holding each of the key driver projections sequentially and observing how the others behaved, resulting in the exclusion of logically inconsistent (or implausible) combination of projections.

**Fig. 8:**
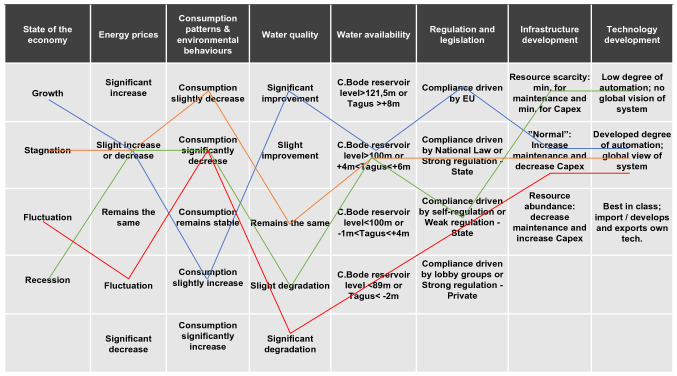
Final set of scenarios: (1) Reference scenario (orange), (2) Strong economic growth scenario (blue), (3) Financial resources’ scenario (green) and (4) Water scarcity scenario (red) (after Luís et al. 2016)

Selection of the scenarios was guided by considering whether each scenario offered a different, though plausible, situation to which the strategic risks can be tested. A synthesis of the cross-consistency analysis resulted in the development of the scenarios, accompanied by a narrative or “storyline” based on the mix of key driver projections: “financial resource scarcity”, “water scarcity” and “strong economic growth”, where each provided a different assumption about future events and developments in the utility’s external business environment (Fig. 8, Table 6).

**Table 6:** Scenario narratives (after Luís et al. 2016)

Scenarios	Narratives
(1) Reference	As Portugal has just exited an economic recession, the state of the economy is becoming stagnant. Energy prices register slight positive or negative fluctuations, and consumption patterns evidence a slight decrease. Both water quality and water availability at source remain at good levels. Water supplied complies with national standards and economic regulation is becoming gradually stronger. Infrastructure developments return to their “normal” configuration, i.e. increasing maintenance and reducing capital investment, thus optimising assets'life without compromising the agreed levels of service to the clients. The company maintains a developed degree of automation, allowing a global view of the system and its centralised operation
(2) Strong economic growth	Significant improvement in water quality happens in a context of strong economic growth. Although existing industries in the water shed increase their activity and new ones arise, they comply with EU water quality legislation and treat all the wastewater before it is discharged into the rivers or the sewage network. Farmers also use permitted pesticides only, complying with the Nitrates Directive. Municipalities' wastewater treatment is of secondary or tertiary levels. There is a slight increase in water consumption. This context of strong economic growth makes way to an increase in Capex, targeting trunk mains' rehabilitation because of their ageing process, and also enables the company to adopt or develop new technology, becoming “best in class”. For example, EPAL augments its own power generation capacity, through the production ofsolar, wind and micro-hydric energy. As a result of all these factors, EPAL faces a reduction in Operational Expenditure, due to reduced costs with energy and chemicals, as well as to an increase in the revenue from the clients
(3) Financial resources’ scarcity	In a prolonged global economic recession context, water quality at sources gets worse, since industries and municipalities cannot afford adequate treatment of the wastewater they produce and, on the other hand, farmers tend to use non-approved pesticides. EPAL faces a significant decrease in consumption, which lowers annual revenue. Both capital and operational expenditures are constrained, andpart of the installed automation system may begin to fail. EPAL moves from a preventive attitude in asset management towards a reactive one.Economic regulation is weak, since regulators know that water utilities have no financial resources either to put measures in place toaccomplish the established levels of service or to pay any fines. Development of new solutions or technology may occur, due to the need to findcheaper ways to operate the water supply system
(4) Water scarcity	Downscaled climate change scenarios indicate that severe drought periods are expected to occur in the next 40 years. During these periods, that may extend over one year or more, there may be a fluctuation in the prices of energy, as energy production is also affected by droughts, as well as a fluctuation in the state of the economy. Consumptions will decrease due to restrictions imposed by EPAL and the regulator. Water quality at sources will also decrease, due to the reduction inflows in the water bodies, which augments the concentration of pollutants.This decrease of water quality may become significant if compliance with environmental standards is self-regulated and economic regulation is weak. In order to cope with the increased water treatment operational costs and the costs associated with the implementation of adaptation measures to water scarcity, along with the reduction in revenue due to a decrease in consumption, tariffs will be gradually increased. EPAL will decrease the regular investment costs, thus increasing maintenance expenditure, and will maintain a developed degree of automation, since having aglobal view of the system is shown to be crucial for its operation in this scenario

*Side by side risks (evolution) comparison* The event-exposure-harm systemic model (Fig. 6) was used as a basis to examine how the likelihood and consequences of the strategic risks behaved in each scenario, where different assumptions are made about the external operating environment of the utility (e.g. increased client revenues in Scenario 2 support investment in technology upgrade compared with resource constraints in Scenario 3 affecting water quality). While the reference scenario is used as the baseline case, we considered the implications of change within the other scenarios (2, 3, 4 in Table 6, Fig. 8) by examining the likelihood of events, and the impacts on the performance of existing control barriers (i.e. both negative and positive influences). A workshop was held involving a select number of experts (*n* = 10) from the baseline assessment for strategic risks (Sect. 3.2.1) to stress-test the risks, guided by a number of questions:

1. How do the baseline risks perform in each scenario? Has the likelihood and consequences of the risks changed? What risks are experiencing the most change in a scenario (or a number of scenarios)?
2. How are existing risk management measures (barriers/controls) performing? What vulnerabilities exist? What opportunities are arising due to good performance?
3. What actions need prioritising, either to safeguard against threats to the strategic objectives or opportunities to maximise the resilience of current risk management measures?

These questions help focus on the potential outcomes of change as opposed to rationalising the change itself (Miller and Waller 2003). This was achieved by asking experts to consider the corporate-level risk exposure, relying on their judgement of the outcomes as having either positive, negative or insignificant implications for the strategic objectives (Table 7). The outcomes were debated and justified, revealing both opportunities and threats to the strategic objectives arising across the scenarios (Koivisto et al. 2009; Defra 2006). At EPAL, this was important to ensure outputs could feed into strategic discussions at Board level.

**Table 7:** Aggregate corporate-level exposure portfolio (from Miller and Waller 2003)

Strategic objectives	Scenario 1	Scenario 2	Scenario ‘n’
Objective 1	+	+	+
Objective 2	−	0	−
Objective ‘n’	−	+	−

Effects on the objectives can be positive (+), negative (−) or insignificant (0)

**Fig. 9:**
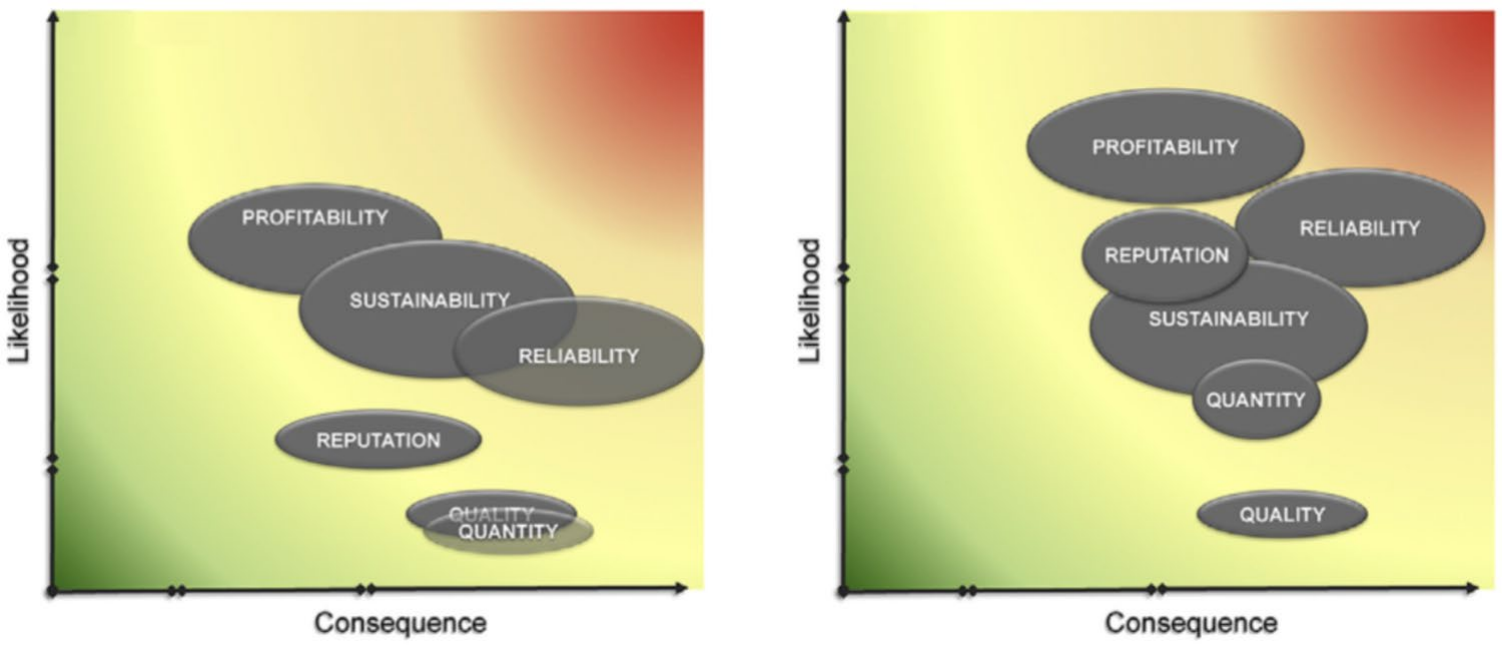
Example of risk ‘heat map’ showing evolved risks (Water scarcity scenario—right) compared to baseline risks (Reference scenario—left)

Outputs from the workshop were synthesised and a narrative of the evolution of baseline risks was presented together with the corresponding risk ‘heat map’ (Fig. 9).

#### Managing multiple, interdependent and dynamic strategic risks

**Fig. 10:**
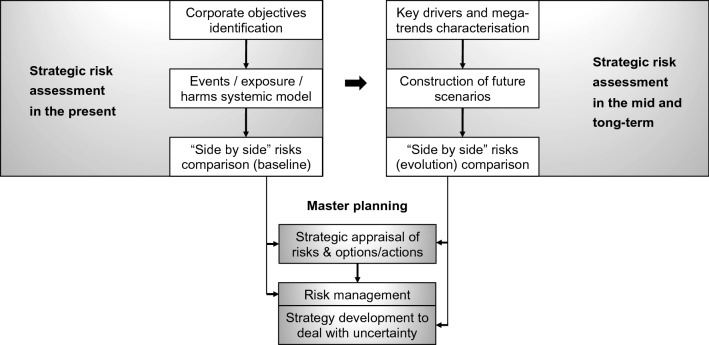
Integrated management of multiple, interdependent strategic risks

EPAL’s long-term extrinsic threats were integrated into the organisation’s strategic risk profile by considering a broader category of risks and their interdependencies. The next step is to ensure the risks are continuously appraised and monitored, through a coordinated management response, thereby improving the ability of the organisation to remain agile and to address both existing risks and emerging threats in their risk management strategy (Fig. 10).

Evolving the risks in a set of scenarios provided insights into the nature of change in the water sector for the next 30 years, building an understanding of the cumulative effects of multiple extrinsic threats on EPAL’s strategic objectives, and their relevance to different business functions for subsequent integration into decision-making. The outcome of stress testing the utility’s strategic risks provided a starting point for the Board to examine what options are worthwhile investing in. Involving both technical and management staff across different business units was critical for building awareness of the impacts of interdependent risks and establishing a case for action for different functional areas. Building a case involved discussions about the overall performance of existing controls. The event-exposure-harm systemic model, accompanying risk narratives and heat maps provided an appropriate level of information for risk managers to communicate the performance of control barriers to the Board, thus providing them with oversight and a process for regular monitoring and review, including:

1. weak and critical failure points of existing control barriers; i.e. those that are likely to fail over the long-term and change the risk profile,
2. sector developments or drivers of change that cause existing barriers to fail,
3. gaps and unintended consequences of the barriers under different scenarios.

The outcome is the ability to review the status of existing barriers and make iterative amendments to improve performance, either by reinforcing the strength of the existing barriers or by implementing new ones. Options can be identified for maintaining the performance of critical barriers, against both short and long-term risks, which will support EPAL in understanding the long-term viability of their portfolio. To ensure existing barriers remain robust, EPAL needs to periodically update the scenarios to consider new trends, emerging issues and associated risks. This is consistent with guidelines in the 2018 ISO 31001 standard that suggests risk management approaches include the use of open systemic models that regularly exchange feedback with the external environment. This step is critical for testing the vulnerability and efficacy of the barriers, which we have suggested could be carried out within one to three years so it feeds into the 10-year periodic review of the master plan at EPAL (Luís et al. 2015).

## Discussion and conclusions

Combining risk and futures methods within a systemic assessment of strategic risks (Fig. 2) represents a significant step forward in the integration of the two fields and evidences the complementarity between the tools employed. The critical strengths of the framework include increasing the utility’s: (a) knowledge of uncertainties, (b) ability to assess the impacts of external developments over long time horizons and possible consequences of actions, and (c) degree of flexibility to adapt to possible future challenges. The benefit for the utility is the ability to be flexible and agile as external conditions change.

The “top-down” and “bottom-up” approach we adopted to assess a utility’s strategic risks enabled operational and strategic risk assessments to be merged, illustrating the interconnectedness of operational, tactical and strategic risks on accessible visuals, such as the event-exposure-harm systemic model (Gormley et al. 2011) and heat maps for “side by side” risk comparisons (Prpich et al. 2011). A key benefit is the ability to capture risk interdependencies; i.e. the individual risk impact on more than one corporate objectives that may cascade from one another, sometimes with positive feedback (Luís et al. 2015). This contrasts with current practices, whereby adaptation measures are proposed and managed in silos, as a response to events triggered by only one single driver, despite being related to other drivers, as noted by Adger et al. (2005). The event-exposure-harm systemic model and risk heat maps produced are important analytical tools that illustrate interdependencies and include supporting narratives that help executives and Board members appraise the multiple threats to the strategic objectives and demonstrate a consistency in handling strategic risks as they are all analysed together.

The long-term nature of risks affecting business performance necessitates taking a systemic view of the water supply system and assessing exposure to strategic risks that are interdependent (ISO 2009; Pollard et al. 2009; Luís et al. 2015). Creating alternative futures, looking at medium and long-term developments, is an important step in improving the flexibility and agility of the organisation to manage uncertainties. The risks utilities face are becoming more external (Chalker et al. 2018). For example, market conditions, customer decisions and demographic changes are occurring irrespective of a utility’s response. External shocks, such as the recent Covid-19 pandemic, have had significant impact on business continuity; for example, social distancing and mobility restrictions caused disruptions to the supply chain and customer services and costly delays in maintenance of infrastructure. We have shown that the baseline strategic risks can be evolved under a set of future utility scenarios (Luís et al. 2016) to reflect on the growing prominence of extrinsic risks, external to utilities and the interconnected nature and complexity of these risks (Allan et al. 2013). This differs from risk assessments based on exposure to isolated risks (e.g. climate change, poor regulatory compliance), which are less effective in considering the interdependencies of strategic risks and managing multiple risks outside of traditional silos.

The inherent difficulty of incorporating stakeholder input in the assessment of strategic risks has been noted (Willis et al. 2004), especially at the strategic level, due to the extent of information and time needed to engage others in discussion of diffuse, long-term strategic issues. We dealt with this by using a similar group of experts from across all levels of the organisation, engaging experts through interviews and stakeholder workshops that bridged traditional organisational and disciplinary silos at EPAL, and encouraged engagement. Engaging Board members from the onset was crucial for securing and sustaining the commitment and involvement of risks experts and senior management throughout the process. Our experience suggests scenario analysis is often useful in encouraging engagement, often through structured discussions, and incorporates the collective intelligence of diverse experts and stakeholders to question and challenge current mindsets about those long-term strategic issues (Henriques et al. 2015; Garnett et al. 2016).

Combing risk and futures methods improves the ability of water utilities to generate integrated forward-looking assessments and insights regarding the dynamics of change, future challenges to the industry and options to manage these. It constitutes a useful tool for strategic/master planning, which may be presented to executives and decision-makers at Board level in a simple and intuitive way, with the benefit that it is based on the solid foundation of the underlying analyses. The approach builds on institutional expertise, promoting the widespread use of risk management within a company whilst unveiling existing knowledge to make it explicit and more useful for the organisation. Linking risk and futures in this way enables managers to evaluate the significance of the risks under different futures, which is useful for prioritisation, monitoring, decision-making and guiding management in an adaptive manner. It contributes to opening up the decision-making process and supports the development of strategies that are more resilient and responsive to external business pressures.

The combination of risk and futures methods, using a ‘semi-quantitative’ approach, allows an assessment of baseline risks over longer-term horizons, thus creating a dynamic process that enable business risks to be viewed as changeable and interconnected, as opposed to static and siloed. A key output of this approach is the identification and prioritisation of ‘events’, ‘exposures’ and ‘harms’, which allows for identifying strategic risks under a given scenario that requires further analysis (including more quantitative risk modelling) in order to reduce epistemic and decision-making uncertainties. In fact, although uncertainty is intrinsic to risk, ideally it should be kept as low as possible. Capturing and communicating the degree of uncertainty implicit in risk assessments is important, because decision-makers (the utility’s Board) need to acknowledge to what extent they can rely on the results, based on which a range of strategies will be implemented, often involving significant investments and costs—and this is the reason why, in the approach described, we used a set of mechanisms, outlined in Sects. 3.3.2 to 3.3.4, to capture and reduce uncertainty.

In this paper, we have unveiled the many similarities between the two fields of risk and futures. We have described how risks and futures combined can provide a holistic, systemic and long-term perspective to improve water utilities’ strategic planning. This approach represents:

- the first systemic analysis of operational, programme-level and corporate risk for EPAL;
- a bottom-up, expert led analysis of risk interdependencies across the utility, addressing aspects as diverse as people, skills and succession planning, the reliability of the asset base, human resources policies and governance structures;
- the basis for long-term, strategic planning under changing conditions of climate, technology, legislation, amongst other megatrends.

Our research has demonstrated the benefits of combining risk and futures in a water utility context. While risk and futures methods are not novel, the sequence in which we combined the methods as well as the participatory nature in which risk experts, risk managers and the Board worked to evaluate the strategic risks is innovative. The steps in the process—summarised in Tables 2 and 4—are structured in a way that allows risk managers to adopt the framework (Fig. 2) for strategic planning in a range of other sectors (e.g. energy, waste). Our continued research in this area is looking at how the framework can be embedded into the strategic planning process of other major sectors. This is a critical step to assess the full potential of the approach, which will leverage the organisation’s ability to deliver value by increasing their business efficiency, sustainability and resilience, through the optimisation of risk reduction options and adaptation measures and the maximisation of opportunities for the short, medium and long-term.
